# The European Trauma Course: Transforming systems through training

**DOI:** 10.1016/j.resplu.2024.100599

**Published:** 2024-03-15

**Authors:** Karl-Christian Thies, Elonka Bergmans, Alistair Billington, Gustavo P. Fraga, Florian Trummer, Ayman O. Nasr, Jonathan Tilsed, Georgie Kamaras, Gregorz Cebula, Alen Protic, Gamal Eldin Abbas Khalifa, Ville Vänni, Souhail Alouini, Katja Kalan Uštar, Paola Perfetti, Ferenc Sari, Diana Cimpoesu, Mary Rose Cassar, Carsten Lott, Lode Blondeel, Fabian Kooij, Elizabete Neutel, Philip Verdonck

**Affiliations:** a EvKB, Dept of Anaesthesia and Critical Care, Bielefeld University Medical Center-Campus Bethel, Bielefeld, Germany; b European Trauma Course Organisation, Niel, Belgium; c Dept of Trauma Surgery, Vera Cruz Hospital-Trauma Center, Faculty of Medicine, University of Campinas, Campinas, Brazil; d European Trauma Course Austria, Vienna, Austria; e Trauma Unit, King Fahad University Hospital & College of Medicine, Imam Abdulrahman Bin Faisal University, Khobar, Saudi Arabia; f Hull York Medical School, United Kingdom; g UEMS Division of Emergency Surgery, European Society for Trauma and Emergency Surgery, United Kingdom; h Luton and Dunstable University Hospital, Luton, United Kingdom; i Jagiellonian University Medical College, Center for Innovative Medical Education, Kraków, Poland; j Department of Anesthesiology, Intensive Medicine and Pain Therapy, University Hospital Rijeka, Rijeka, Croatia; k Emergency and Disaster Medicine, Egyptian Resuscitation Council, Egypt; l Helsinki Trauma Unit, Helsinki, Finland; m Tunesian Resuscitation Council, Sousse, Tunisia; n Dept of Anaesthesia and Critical Care, Trbovlje General Hospital, Trbovlje, Slovenia; o Emergency Department at Latisana, Azienda Sanitaria Universitaria Friuli Centrale, Italy; p Department of Emergency Medicine, Skellefteå Hospital, Region Västerbotten, Sweden; q University of Medicine and Pharmacy “Grigore T. Popa”, Emergency Medicine, II-nd Surgery Department, Hospital “Sf. Spiridon” Iasi, Romania; r Emergency Department, Mater Dei Hospital, Malta, University of Malta, Malta; s Klinik für Anästhesiologie, Universitätsmedizin Mainz, Mainz, Germany; t Belgian Defense, Belgium; u Anesthesiologie Amsterdam UMC, locatie AMC, Amsterdam Zuidoost, Netherlands; v Department of Anaesthesiology, Intensive Care Medicine and Emergency. Unidade Local de Saúde de Santo António, Porto, Portugal; w Emergency Departement, Antwerp University Hospital, Faculty of Medicine and Health Sciences, University of Antwerp, Belgium

**Keywords:** Trauma, Allied Health Professionals, European Trauma Course, ETC, ATLS, Team Training

## Abstract

The European Trauma Course (ETC) exemplifies an innovative approach to multispecialty trauma education. This initiative was started as a collaborative effort among the European Society for Emergency Medicine, the European Society for Trauma and Emergency Surgery, and the European Society of Anaesthesiology under the auspices of the European Resuscitation Council. With the robust support of these societies, the project has evolved into the independent European Trauma Course Organisation.

Over the past 15 years, the ETC has transcended traditional training by integrating team dynamics and non-technical skills into a scenario-based simulation course, helping to shape trauma care practice and education. A distinctive feature of the ETC is its training of doctors and allied healthcare professionals, fostering a collaborative and holistic approach to trauma care. The ETC stands out for its unique team-teaching approach, which has gained widespread recognition as the standard for in-hospital trauma care training not only in Europe but also beyond. Since its inception ETC has expanded geographically from Finland to Sudan and from Brazil to the Emirates, training nearly 20,000 healthcare professionals and shaping trauma care practice and education across 25 countries. Experiencing exponential growth, the ETC continues to evolve, reflecting its unmet demand in trauma team education.

This review examines the evolution of the ETC, its innovative team-teaching methodology, national implementation strategies, current status, and future challenges. It highlights its impact on trauma care, team training, and the effect on other life support courses in various countries.

## Background

Major trauma remains a significant healthcare challenge and constitutes 10% of the global disease burden.^1^ The delivery of high-quality trauma care is pivotal, with the potential to markedly reduce mortality following severe injuries.^2^

Clinical trauma management constitutes a comprehensive ‘chain of care' that commences at the injury scene, traverses prehospital systems, and extends through in-hospital resuscitation and definitive treatment. This pathway concludes with rehabilitation aimed at a full reintegration into daily life.

Within the trajectory of trauma care, in-hospital trauma teams play a key role as they take charge of patient management from prehospital services. Despite the relatively brief duration spent in the resuscitation room, this period is marked by crucial decision-making, the implementation of critical interventions, the establishment of treatment priorities, and the definition of a personalised patient pathway, all of which profoundly affect patient outcomes. Within this time-sensitive environment, various processes unfold concurrently and are managed by diverse team members with complementary skill sets. Remarkably, these teams, tasked with making critical decisions, often convene just before patient arrival and may not have collaborated previously. This highlights the intricate nature of trauma care and emphasises the need for effective teamwork, coordination, and decision-making within compressed timeframes.

Introduced in 2008, the European Trauma Course (ETC) emerged as a training program designed for multispecialty trauma teams involved in acute care. The core mission of the ETC is to decrease mortality, morbidity, and disability secondary to major trauma through high-quality, multidisciplinary, and multiprofessional trauma training. Over 15 years, the ETC has experienced substantial growth, expanding its reach to 82 centres across 25 countries and conducting a total of 133 courses in 2022.

As the ETC marks its 15th anniversary, it serves as an opportune moment for reflection on the course's evolution and to contemplate the future of trauma team training.

## Development of the programme

The vision for a team-focused trauma life support programme emerged in 2002 during a meeting with international experts in Bologna, Italy.^3^ The initiative gained momentum in 2004 when the European Resuscitation Council (ERC) embraced it, establishing the European Trauma Working Group (ETWG) with support from the European Society of Anaesthesiology (ESA), the European Society for Trauma and Emergency Surgery (ESTES), and the European Society for Emergency Medicine (EuSEM).^4^ The ETWG identified the need for a multispecialty trauma team training programme across various European countries.

The development of the European Trauma Course (ETC) unfolded over a 2-year period, building upon a pre-existing Portuguese trauma course. Following four pilot courses in Malta (twice) and Norway and Italy,^5^ the ETC was officially launched in 2008 at an annual ERC conference in Belgium.^6^ This milestone marked the beginning of a program aimed at enhancing trauma care through comprehensive team-focused training.

## Course concept

The ETC is open to all healthcare staff regularly involved in the initial reception of major trauma patients and spans over 2.5 days.

Exemplifying interdisciplinary integration, the faculty comprises various medical specialities, which fosters robust discussions across advanced major trauma care domains, an aspect frequently highlighted by participants. Another distinctive feature of the ETC is its horizontal approach to trauma management, in which team members complete tasks in parallel, mirroring the collaborative workflow of trauma teams in a major trauma resuscitation bay.

The course transitioned from traditional didactic teaching to simulation-based training, focusing on teamwork and non-technical skills (NTS).^7^ Candidates undergo training in small groups, dedicating 85% of the course to hands-on participation in nine workshops (Table 1), complemented by two lectures and one demonstration.

**Table 1:** The ETC comprises of nine workshops covering all aspects of major trauma including multi-trauma, trauma in pregnancy and geriatric trauma.

**ETC Workshops**
Airway trauma
Thoracic trauma
Shock
Head trauma
Spinal trauma
Abdominal trauma
Paediatric trauma
Extremity trauma
Transfer

**Fig. 1:**
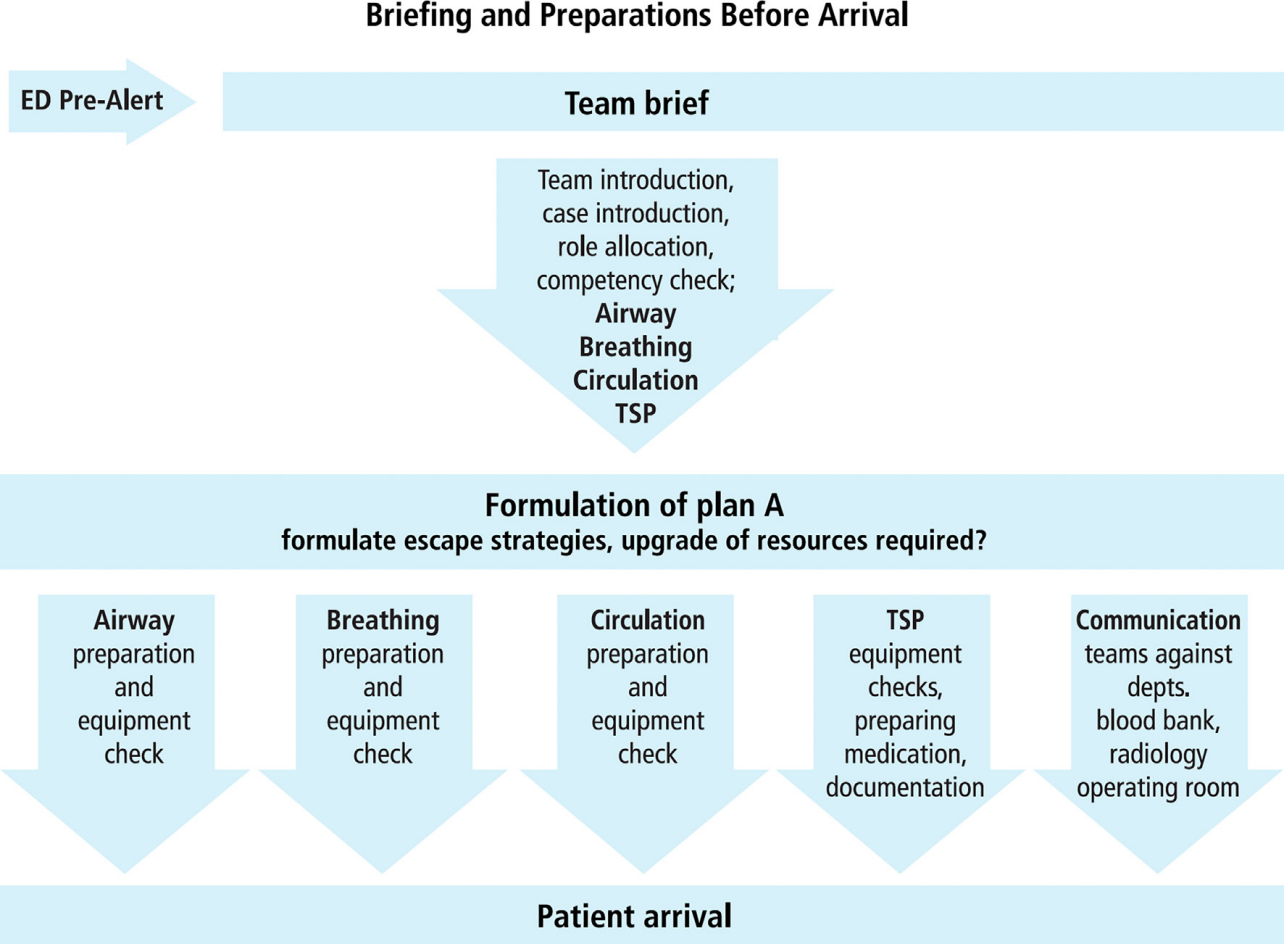
**Deconstruction of a scenario A:** All scenarios follow a similar workflow: The team leader shares information from ambulance control about the expected patient with the entire trauma team. This briefing ensures all team members are informed and prepared. Then the competences of each team member are established and their corresponding roles in the team are confirmed. Subsequently, all team members perform thorough checks of their respective equipment. At the same time further resources are mobilized if required.

**Fig. 2:**
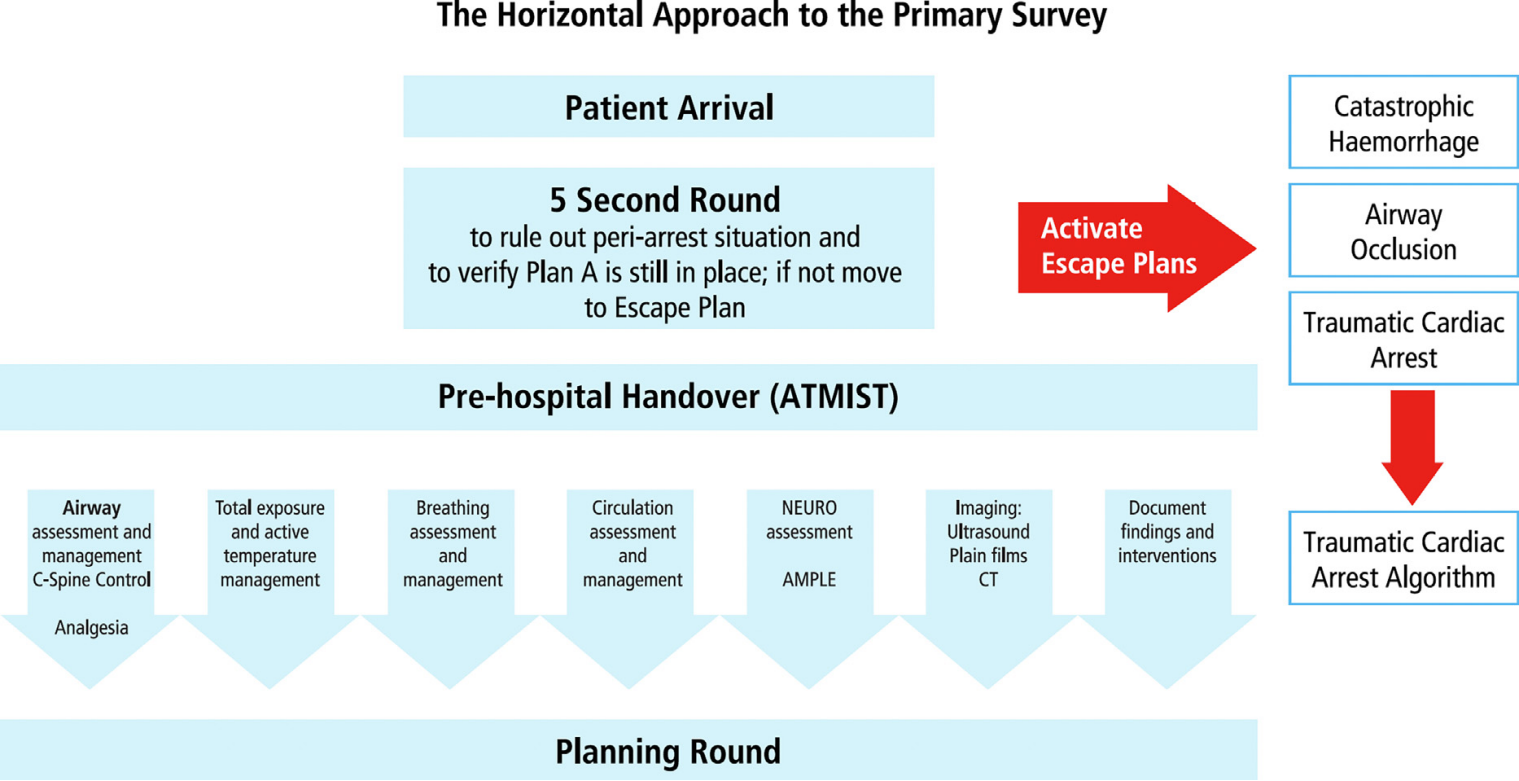
**Deconstruction of a scenario B:** Upon patient arrival, the team leader performs a swift ‘5 second round’ to identify any immediate life-threatening conditions. This is followed by the team attentively receiving the handover. The primary survey then proceeds in a parallel manner, with team members simultaneously assessing and treating the patient. If serious and unexpected problems are encountered, team members or the leader can initiate a ‘STOP procedure’. This ensures immediate team-wide awareness of the changing situation and alignment of priorities.

**Fig. 3:**
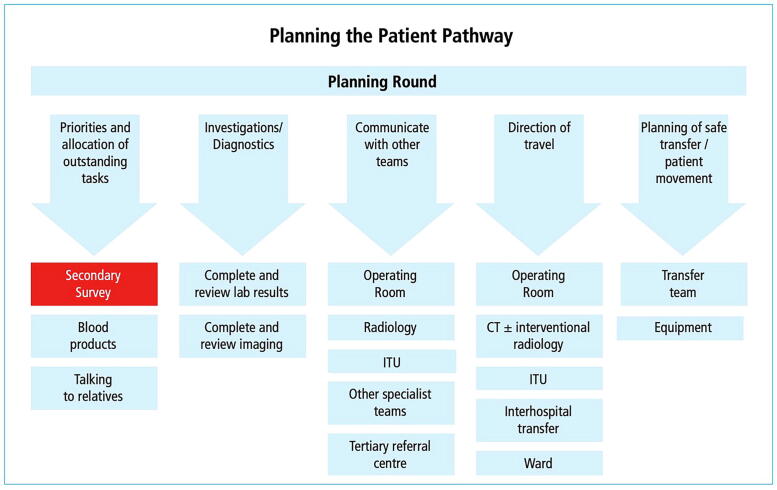
**Deconstruction of a scenario C:** The scenario concludes with a summary and planning round, which serves to collate all findings, review the measures taken so far, and establish an individual patient pathway. Several factors influence this pathway. Subsequently, the planning round transitions into the debriefing of the scenario.

Each workshop comprises three or four scenario-based modules, each with specific learning outcomes such as 'recognition and treatment of hypovolemic shock' or 'performance of a focused neurological assessment in a trauma patient’. The anatomy of a standard ETC scenario is depicted in Fig. 1, Fig. 2, Fig. 3. Modules may also include teaching technical and cognitive skills, such as chest drain insertion or a structured interpretation of radiological imaging.

The modular concept offers significant flexibility, allowing adaptation to different local needs without altering the core learning outcomes. For instance, variations in spinal immobilisation practices can be accommodated.

Following the clinical simulation scenarios, a debriefing session is conducted. Each workshop requires a minimum of two faculty members to effectively manage the challenges of dynamic team-based scenarios and to conduct meaningful and insightful debriefings.

The candidates are continuously assessed throughout the course with respect to their clinical skills and abilities as team members and leaders. On the final day, each candidate participates in a standardised summative assessment to evaluate team leadership, clinical management, and practical skills.

## Focus on non-technical skills

The typical trauma team is a diverse group of clinicians from various specialities, and professionals from different fields, such as nursing, operating department staff, and radiography. These multiprofessional and multispecialty teams often come together on short notice, emphasising the need for a predefined team structure with allocated roles, goal-directed communication, and clear leadership to ensure effective, safe, and timely performance.

The ETC developed a formalised framework to enable newly formed teams to operate optimally. This framework draws from 15 Crew Resource Management (CRM) principles described by Rall^8^ and the anaesthetists’ NTS assessment framework outlined by Flin et al.^9^. This structured approach is applied to all scenarios in the course, allowing candidates to experience enhanced teamwork through this systematic framework while simultaneously practising their NTS. During scenario debriefs, candidates are encouraged to focus on team dynamics and individual NTS in a supportive manner. Initially led primarily by instructors on day 1, candidates progressively assume a more active role in the debrief sessions as they gain confidence. By the end of the course, candidates can lead debriefs, analyse team dynamics, identify best practices, and pinpoint areas for improvement, all conducted in a respectful manner to maintain psychological safety, overseen by instructors. This methodology ensures a comprehensive and constructive learning experience for participants.

## Educational background

### Educational theory

The success of the ETC can be partly attributed to its innovative team-teaching approach, grounded in the principles of adult learning.^10^

Acknowledging the limitations of traditional audio-visual teaching methods in terms of learning retention,^11^, ^12^ ETC has transitioned from didactic teaching towards immersive scenario-based simulations aligning with experiential learning principles.^13^ Before attending the course, participants are encouraged to acquire robust knowledge of trauma management by engaging them with a comprehensive course manual written by experts across relevant trauma care specialities.^14^ While studying the manual is required for preparation, the course avoids formal knowledge assessments, aligning with the principle of internal motivation, which is prerequisite for effective adult learning.^15^

Each of the 30 scenario-based modules of the course represents (Table 2) an experiential learning cycle.^16^ Participants apply a predetermined concept of trauma care^17^ during the scenario. Reflection on experiences in the scenario during the debriefing phase leads to the confirmation or modification of pre-existing concepts, aligning with Kolb's model.^18^ In addition, one participant is assigned to observe the scenario and share thoughts about the NTS observations during debriefing, embracing the advantages of observational learning.^19^

**Table 2:** This table outlines the structure of a generic ETC learning module based on a scenario-driven approach.

**Composition of a Learning Module**
• Introduction to the learning objectives
• Scenario
o Team brief
o 5-second round
o Handover
o Primary survey
o STOP procedure
o Planning round
• Debrief

Thoughtful debriefing is crucial to emphasise learning points, that may vary among participants due to diverse backgrounds, experiences, and team dynamics. The ETC enhances team-focused debriefing through a “learning conversation” technique, rooted in authentic andragogical principles,^20^, ^21^ which allows exploration of issues that the team wish to reflect upon, whilst the instructors facilitate a positive and non-threatening learning environment, promoting open expression and acceptance of positive and negative critiques. This ensures a constructive and tailored learning experience that conforms to the principles of adult learning and simulation-based education.

### Simulation technique

Authentic simulation of a trauma team experience presents challenges.^10^ The ETC has recognised the effectiveness of allowing uninterrupted teamwork during scenarios where team dynamics closely mirror real-life situations, creating a genuinely immersive resuscitation experience even with low-fidelity simulation. Notably, this sets the ETC apart from other life support courses where interruptions are common in providing the team with additional information.

To maintain realism while delivering essential information to the team, the ETC employs several techniques that make instructors 'invisible':

• Role Cards: Each team member received a role card detailing their initial assessment findings. They must convey this information to the team leader after completing their assessment.
• Vital Signs Monitor with Remote Control: A vital signs monitor with a remote control is utilised to present and adapt vital signs during the scenario.
• Instructor Integration: Instructors are sometimes integrated into the team, allowing them to provide additional information during the scenario or demonstrate skills in a clinical context, aligning with the concept of situated learning.^22^
• Whispered Information: If additional information is required to guide the scenario towards the intended learning outcome, a second instructor outside the team will discreetly whisper the necessary details to a specific team member. Team members must convey this new information to the team at an appropriate time.

These techniques not only maintain the integrity of the simulation but also enhance the authenticity of the learning experience by seamlessly integrating essential information into the team's workflow.

### Instructor training

Course faculties select potential instructors based on specific criteria, requiring evidence of educational experience (e.g., GIC) for their inclusion as instructor candidates. A compulsory instructor day, focusing on the programme's educational aspects, precedes each course. Candidates must successfully participate in three ETC courses to become full instructors, demonstrating proficiency in delivering simulation scenarios, employing a team-based approach, and conducting effective debriefs. During the instructor day, the faculty receives updates on educational best practices, engages in workshops, and practices clinical scenarios, including coaching and assessment techniques. This ensures the instructor team is well-prepared, unified, and aligned with ETC principles.

## Evolution of the programme from 2008 to 2023

ETC has evolved through improvements, refinements, and new developments based on quantitative and qualitative feedback from participants, instructors and observers. The ETWG has matured into the independent European Trauma Course Organisation (ETCO), an international not for profit organisation based in Belgium.^4^ To ensure ongoing collaboration with the founding organisations all of them are represented on the board of the ETCO, actively involved in updating the educational content of the programme to ensure ETC remains at the leading edge of current practice. National chapters of the ETC serve a supportive role at regional level, while the governance of the programme is centralised at the ETCO head office.

### Evidence-based trauma management updates

The course content, anchored in the course manual, undergoes a meticulous update cycle every four to five years, ensuring it remains current with advances in trauma care. Authored by experts identified through an extensive literature review, the manual's revisions are vetted by ETC course directors and ratified by the founding societies to maintain the highest quality and relevance. The near release of the fifth edition of the ETC manual underscores the rapid evolution in evidence-based trauma care over the past 15 years. Transiting from the initial recommendation of 2 L crystalloid for fluid resuscitation, the current standard embraces haemostatic resuscitation and major haemorrhage protocols. Notably, the revised priorities for patient immobilisation were aligned with contemporary best practices. Evidence-based approaches, such as tranexamic acid, the traumatic cardiac arrest algorithm, and early whole-body CT, have also been incorporated. Moreover, the recognition of the efficacy of major trauma networks, which are increasingly standard in Europe, further acknowledges the need for optimised, interconnected care pathways.

### Educational updates

The workshop scenarios and associated teaching materials underwent incremental updates, with more patient comorbidities, peri-arrest situations, traumatic cardiac arrest, and scenarios involving older patients. Along with content updates, the simulation support materials were improved, with tablets used as vital function monitors, blood gas printouts, scene pictures, and patient images.

In 2012, a session focusing on the NTS was introduced. This detailed focus on NTS in a life support course was innovative and well-received by the participants. Given that the participants’ understanding of the NTS has grown over time, the NTS session was updated to provide more in-depth coverage of the topic; however, huge international variations remain in the understanding of the NTS.

A discussion session on trauma networks and local transfer arrangements was transformed into a challenging simulation scenario, vividly illustrating the need for several levels of communication and the involvement of multiple team members if transfer decisions and arrangements must be made while simultaneously dealing with a time-critical injuries.

### Trauma Support Practitioner programme

Initially designed for physicians and highly trained advanced care practitioners, the entry criteria for the ETC have been subsequently expanded to include regular allied health practitioners, such as nurses, operating department practitioners, and paramedics, who play crucial roles in the care of major trauma patients. However, feedback from these participants revealed a disparity in the expected learning outcomes. The assumption that allied health professionals should achieve identical learning outcomes as physicians resulted in scenarios that resembled role-playing rather than authentic simulations. This created additional stress for allied health care practitioners, particularly when placed in unrealistic roles, like assuming the team leader position.

As a remedy to this challenge, the Trauma Support Practitioner (TSP) was introduced in 2014 to address this issue. TSPs have their own sets of learning outcomes and assessments tailored to match the responsibilities of allied healthcare professionals. Pilot programs in the UK, Netherlands, and Germany demonstrated that the addition of one or more TSPs to a team significantly enhanced the realism of training for all professions involved. This approach was well-received by the participants and proved to be feasible. Consequently, the TSP’s role was formally adopted in 2022, ensuring that the ETC provides a more authentic and inclusive training experience for a diverse array of healthcare professionals involved in major trauma care.

### E-learning recertification course

Following the ETC's successful launch, a significant demand for recertification arose. To address this, a specialised recertification program was developed, initially planned as a one-day in-person course. However, due to the COVID-19 pandemic, it evolved into an online e-learning format, officially launched in 2021, providing a flexible and accessible recertification option. This recertification course adopts a scenario-based approach and culminates with a comprehensive knowledge test. Currently, nearly 1300 candidates have completed the course, and the feedback received has been markedly positive. Many participants expressed a preference for online recertification over repeated face-to-face courses (data awaiting publication).

### International growth of the programme

Since its official launch in 2008, national ETC programmes have been established in 25 countries, including Europe, Asia, North Africa, and South America (Table 3). Currently, there are 19 countries on the waiting list to establish national ETC programmes.

**Table 3:** This table provides an overview of the start date of the European Trauma Course (ETC) in each country, detailing the number of active course centers and the total candidates trained up to August 2023.

Country	Number of courses	Participants (total)	First course	Current course centres
Austria	103	1788	2008	1
Belgium	81	1156	2008	8
Brazil	6	72	2019	1
Croatia	30	358	2010	1
Cyprus	0	0		1
Denmark	15	264	2013	1
Egypt	98	1591	2009	3
Finland	32	712	2011	4
Germany	184	3155	2008	17
Greece	2	23	2015	1
Hungary	18	342	2014	2
Ireland	3	59	2012	1
Italy	50	940	2007	1
Jordan	6	58	2015	1
Malta	19	366	2006	1
Netherlands	13	142	2011	2
Norway	1	21	2007	1
Poland	31	446	2009	3
Portugal	58	957	2009	4
Romania	14	214	2014	3
Saudi Arabia	5	67	2013	1
Slovenia	20	225	2011	1
Spain	10	76	2019	3
Sudan	35	447	2013	2
Sweden	19	173	2010	1
Switzerland	7	95	2014	1
Tunisia	13	270	2016	3
UAE	36	407	2015	2
UK	217	4307	2009	21

**Fig. 4:**
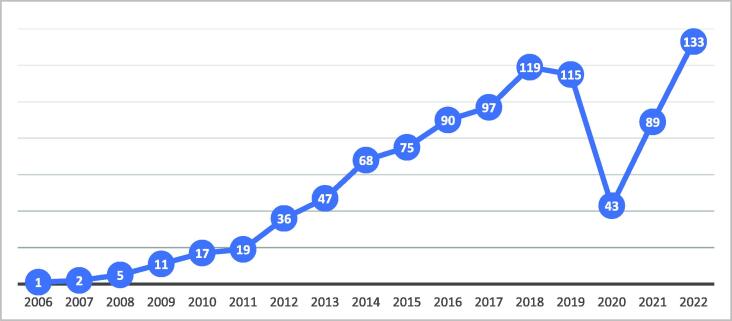
This graph illustrates the rapid growth rates of the ETC programme as well as the significant decrease in the number of courses offered during the pandemic period. This emphasises the urgent need for adopting innovative teaching methodologies to enhance resilience and adaptability of educational programmes in response to unforeseen challenges.

The ETC experienced exponential growth in the number of courses offered annually, underlining the significant unmet demand for advanced and multidisciplinary trauma team training (Fig. 4). Geographically, the programme extends from Brazil to the Emirates and from Finland to Sudan, demonstrating its global impact and influence in diverse regions.

### Implementation in individual countries

The adoption of the ETC is dependent on the organisational structures within trauma care, particularly the specialities involved in initial trauma management in the resuscitation room. Countries with established emergency medicine specialities exhibit a high adoption rate, whereas those relying on surgical specialities often show lower adoption rates, given the longstanding prevalence of Advanced Trauma Life Support (ATLS) in training curricula. Many countries have incorporated ETC into accredited trauma training for emergency medicine, anaesthesia, intensive care, and surgery.

Implementation strategies vary across countries. Some run all courses within a centralised ETC centre, while others opt for multiple centres or deploy mobile ETC at various clinical sites.

Notably, nearly every country has trained a local cohort of instructors, enabling courses to be conducted in native languages. This has proven to be particularly beneficial for fostering in-depth discussions on non-technical skills.

In terms of participant composition, course candidates are mostly from emergency medicine, anaesthesia, or intensive care, while the participation rates of surgical specialities and allied health professionals are lower.

Many countries have participated in pilot projects for course improvement, such as the TSP project, the development of new scenarios, and the implementation of technology-enhanced learning.

### Effect of the programme

The ETC has significantly influenced trauma education and systemic approaches to trauma care at national and international level. Its principles have been effectively integrated into clinical practices across many hospitals, demonstrating the practical applicability and relevance of the ETC's methodologies. This transition from an educational model to clinical application marks a significant evolution in trauma care, reflecting the ETC's role in enhancing trauma management practices.

Central to this evolution is the ETC's team-teaching methodology, which represents a notable advancement in life support training. This methodology has been incorporated into the European Resuscitation Council (ERC) course framework and training programmes, setting new standards for life support courses.^23^ Moreover, specific team-oriented strategies, such as the pre-patient arrival briefing, have been codified into clinical protocols as the 'Zero-Point Survey'.^24^ This approach, critical for team preparation, has transformed the way in which teams respond to trauma cases.

Thus, the ETC's contribution transcends educational boundaries, contributing to a methodical shift in trauma care. It promotes inter-professional collaboration, leading to improved coordination among healthcare professionals and enhanced patient outcomes. The ETC's systematic and innovative approach has played a key role in redefining trauma care delivery internationally.

The Additional File 1 provides a summary of the implementation strategies adopted by individual countries and the effect of the programme at national levels.

### Future of the programme

The international success of the ETC highlights its crucial role in filling a longstanding gap in trauma training. However, its overwhelming demand surpasses the course availability. Expanding an educational programme globally results in various challenges and necessitates careful examination to maintain its integrity.

In this section, we outline some future challenges of the ETC and the strategies implemented to tackle them.

### Accessibility

The extended duration of the ETC, requiring 2.5 days for candidates and 3.5 days for instructors, poses challenges in the context of global staff shortages and financial constraints. Currently, securing the necessary time off for both participants and instructors has become increasingly difficult. Striking a balance between programme accessibility and maintaining high standards is a key priority, especially considering that further expansion depends on the availability of well-trained instructor cohorts.

One solution involves leveraging technology-enhanced learning, as demonstrated by the successful e-learning recertification course. Interactive pre-course e-learning aligns with the preferences of the digitally proficient generations,^25^, ^26^ offering self-directed and flexible learning.^27^ Transferring parts of trauma-related motor skill teaching to a virtual environment enhances efficiency and reduces the time required for face-to-face instruction.^28^ Innovative methods such as serious gaming bridge the gap between theoretical knowledge and practical skills and provide immersive experiences in real-life trauma scenarios.^29^ The emergence of virtual reality (VR) in medical education presents opportunities for team-based trauma training in risk-free environments.^30^

The ETC anticipates that embracing these technologies will enhance learning efficiency and teaching resilience. It recognises digital solutions not as substitutes but as enhancements to traditional methods, alleviating demands on instructors and reducing face-to-face time.

### Quality assurance

Maintaining consistent quality, defined by its ‘fitness for purpose' and ‘value for money',^31^ across diverse nations is challenging. It is necessary to ensure that educational content, delivery methods, and assessment standards meet uniformly high-quality levels.

#### Standardisation versus local diversity

While one of the strengths of the ETC is its adaptability to meet local needs, there is a fine line between customisation and the dilution of core educational content.^32^ Key competencies must be consistently attained across all venues, irrespective of local adaptations. Therefore, all local adaptations require approval by the ETC educational team to ensure that key competencies remain unchanged.

#### Expanding the instructor cohort

Efforts to shorten the course and expand instructional capacities must not compromise the thorough training of faculty and preserve the consistent delivery of high-quality education. Recognising this challenge, a dedicated working group was assigned to develop a robust faculty development programme. The programme incorporates various elements, including the integration of e-learning platforms. These platforms will play a crucial role in maintaining teaching quality, ensuring consistency, and facilitating seamless exchange of information among instructors. By prioritising comprehensive faculty development, the program aims to uphold its commitment to excellence in trauma training delivery.

#### Keeping scientifically up-to-date

Recognising the dynamic nature of medicine, the ETC manual is regularly reviewed by international trauma expert groups to ensure that updates incorporate the latest advancements in trauma care. These updates were seamlessly integrated into teaching materials to ensure consistent and up-to-date content delivery across all venues.

### International expansions

Global expansion demands a strategic balance in resource allocation, considering staffing, infrastructure, and technological variations exist across different regions. Successful program implementation depends on the collaborative efforts of national institutions, educators, and stakeholders. Infrastructure, including quality training centres and necessary equipment, plays a crucial role, with new ETC centres being required to meet specific criteria for recognition. This scrutiny is pivotal when expanding to countries with limited medical resources. Importantly, the successful implementation of the ETC in non-European countries underscores its adaptability and commitment to addressing the unique challenges faced by diverse healthcare environments.

### Language barriers

Language differences hinder effective communication and understanding. Ensuring language proficiency among instructors and participants is vital for a successful global expansion. Therefore, there is a focus on rapidly training local faculty members to conduct courses in their native language.

While having course materials in local languages is advantageous, the process of translating and updating these materials is resource-intensive. Ensuring that translations capture the essence and nuances of the original content presents a significant challenge.

### Economic disparities

There is a recognised desire to adopt the programme in low-income countries. To ensure that the course remains financially sustainable, the ETC operates a staggered fee system aligned with the general income levels of its countries. This approach allows low-income countries to pay a fraction of the fees charged in high-income nations, which ensures broader accessibility and affordability while preserving the economic viability of the programme.

In regions with limited technological resources or inconsistent access to the Internet, all courses and educational materials were provided in paper format.

### Political instability and conflict zones

Adapting to and mitigating the impact of political changes on a programme’s operations poses a significant challenge to global expansion. The situations in regions such as Sudan and Ukraine stress the unique challenges in certain areas. While the demand for training is high in conflict zones, maintaining existing programmes and establishing new ones is challenging.^33^ The unpredictability and dangers associated with conflict zones make it difficult to ensure the consistent presence and safety of instructors. Web-based solutions might serve as a viable alternative, helping to bridge the training gap without requiring instructors to physically venture into high-risk areas. Thus, by leveraging digital platforms, ETC may continue to impart vital knowledge even in challenging circumstances.

### Quality improvement

Ensuring a programme's ongoing effectiveness and adaptability to evolving global needs requires feedback mechanisms and continuous research. Immediate feedback from candidates and instructors reflects their satisfaction, engagement, and perceptions of course content and delivery. Summative tests assessed participants' grasp of the principles and practices taught.

The ETC aims to enhance its evaluation strategy by assessing the on-the-job application of ETC principles.^34^ Additionally, evaluating the impact on broader organisational structures and clinical outcomes, such as morbidity and mortality rates, is a future goal. Developing robust evaluation methods for this impact, especially in dynamic trauma care settings, poses challenges but is essential for comprehensive program assessment.

## Conclusion

The ETC provides simulation-based, interdisciplinary, and multiprofessional trauma training with a strong emphasis on teamwork and NTS. It distinguishes itself as the only internationally accredited trauma course that trains doctors and allied healthcare professionals together in multispecialty teams. By doing so, the ETC is not just educating medical professionals but actively transforming practice in trauma care. Adapting to the changing landscape of trauma care and its challenges over the past 15 years, the ETC remains committed to continuing this proactive and transformative approach, further shaping and enhancing global trauma care systems.

## Availability of data and materials

All data will be made available upon reasonable request.

## CRediT authorship contribution statement

**Karl-Christian Thies:** Writing – review & editing, Writing – original draft, Supervision, Project administration, Investigation, Conceptualization. **Elonka Bergmans:** Writing – review & editing, Writing – original draft, Supervision, Methodology, Investigation, Conceptualization. **Alistair Billington:** Writing – review & editing, Writing – original draft, Investigation, Conceptualization. **Gustavo P. Fraga:** Writing – review & editing, Writing – original draft, Investigation. **Florian Trummer:** Writing – review & editing, Writing – original draft, Investigation. **Ayman O. Nasr:** Writing – review & editing, Writing – original draft, Investigation. **Jonathan Tilsed:** Writing – review & editing, Writing – original draft, Investigation. **Georgie Kamaras:** Writing – review & editing, Writing – original draft, Investigation. **Gregorz Cebula:** Writing – review & editing, Writing – original draft, Investigation. **Alen Protic:** Writing – review & editing, Writing – original draft, Investigation. **Gamal Eldin Abbas Khalifa:** Writing – review & editing, Writing – original draft, Investigation. **Ville Vänni:** Writing – review & editing, Writing – original draft, Investigation. **Souhail Alouini:** Writing – review & editing, Writing – original draft, Investigation. **Katja Kalan Uštar:** Writing – review & editing, Writing – original draft, Investigation. **Paola Perfetti:** Writing – review & editing, Writing – original draft, Investigation. **Ferenc Sari:** Writing – review & editing, Writing – original draft, Investigation. **Diana Cimpoesu:** Writing – review & editing, Writing – original draft, Investigation. **Mary Rose Cassar:** Writing – review & editing, Writing – original draft, Investigation. **Carsten Lott:** Writing – review & editing, Writing – original draft, Investigation. **Lode Blondeel:** Writing – review & editing, Writing – original draft, Investigation. **Fabian Kooij:** Writing – review & editing, Writing – original draft, Investigation. **Elizabete Neutel:** Writing – review & editing, Writing – original draft, Investigation. **Philip Verdonck:** Writing – review & editing.

## Declaration of competing interest

The authors declare that they have no known competing financial interests or personal relationships that could have appeared to influence the work reported in this paper.

## References

[1] Haagsma J.A., Graetz N., Bolliger I. (2016). The global burden of injury: incidence, mortality, disability-adjusted life years and time trends from the Global Burden of Disease study 2013. Inj Prev.

[2] Moran C.G., Lecky F., Bouamra O. (2018). Changing the system-major trauma patients and their outcomes in the NHS (England) 2008–17. EClinicalMedicine.

[3] Araujo R, Della Corte F et al. ERC, EUSEM, ITACCS, BTA and ESICM. European comprehensive training course on prehospital advanced trauma life support in adults. Eur J Emerg Med. 2002;9:280-2. 10.1097/00063110-200209000-00016. PMID: 12394630. 12394630

[4] Thies K.C., Deakin C.D., Lott C. (2014). The European trauma course–trauma teaching goes European. Resuscitation.

[5] Thies K., Gwinnutt C., Driscoll P. (2007). The European Trauma Course–from concept to course. Resuscitation.

[6] Lott C., Araujo R., Cassar M.R. (2009). The European Trauma Course (ETC) and the team approach: past, present and future. Resuscitation.

[7] Fletcher C.G.L., McGeorge P., Flin R.H. (2002). The role of non-technical skills in anaesthesia: a review of current literature. BJA.

[8] Rall M., Howard S.K., Dieckmann P., Miller R.D., Eriksson L.I., Fleisher L.A., Weiner-Kronish J.P., Young W.L. (2015). Milleŕs Anesthesia.

[9] Flin R., Patey R., Glavin R., Maran N. (2010). Anaesthetists' non-technical skills. BJA.

[10] Davis M., Cassar M.R., Driscoll P. (2011). The European Trauma Course: using experience to refine an educational initiative. Trauma.

[11] Donoghue G.M., Hattie J.A.C. (2021). A Meta-Analysis of Ten Learning Techniques. Front Educ.

[12] Graham K.L., Cohen A., Reynolds E.E., Huang G.C. (2019). Effect of a flipped classroom on knowledge acquisition and retention in an internal medicine residency program. J Grad Med Educ.

[13] Kolb D.A. (1984).

[14] Thies K, Mountain A, Goode P. European Trauma Course – The Team Approach. 4th ed.; 2020. https://www.europeantraumacourse.com.

[15] Knowles M.S. (1980).

[16] Fewster-Thuente L., Batteson T.J. (2018). Kolb's experiential learning theory as a theoretical underpinning for interprofessional education. J Allied Health..

[17] Williams D.E. (2016). The future of medical education: flipping the classroom and education technology. Ochsner J.

[18] Kolb D.A. (2015).

[19] O'Regan S., Molloy E., Watterson L., Nestel D. (2016). Observer roles that optimise learning in healthcare simulation education: a systematic review. Adv Simul (Lond).

[20] Norris E.M., Bullock I. (2017). A ‘Learning conversation’ as a style of feedback. MedEdPublish.

[21] Davis M., Denning K. (2018). Listening through the learning conversation: a thought provoking intervention. MedEdPublish.

[22] Lave J., Wenger E. (1991).

[23] Michael M., Biermann H., Gröning I. (2022). Development of the Interdisciplinary and Interprofessional Course Concept “Advanced Critical Illness Life Support”. Front Med (Lausanne).

[24] Reid C., Brindley P., Hicks C. (2018). Zero point survey: a multidisciplinary idea to STEP UP resuscitation effectiveness. Clin Exp Emerg Med.

[25] Guze P. (2015). Using technology to meet the challenges of medical education. Trans Am Clin Climatol Assoc.

[26] Prensky M. (2001). Digital natives, digital immigrants. On the Horizon.

[27] Phillips J., Wiesbauer F. (2022). The flipped classroom in medical education: a new standard in teaching. Trends Anaesth Crit Care.

[28] Bergmans E., Billington A., Thies K.C. (2023). From tradition to innovation: a comparison of the traditional 4-step approach versus a blended learning modification for technical skills teaching. Scand J Trauma Resusc Emerg Med.

[29] Kim S., Song K., Lockee B., Burton J. (2018).

[30] Issleib M., Kromer A., Pinnschmidt H.O. (2021). Virtual reality as a teaching method for resuscitation training in undergraduate first year medical students: a randomized controlled trial. Scand J Trauma Resusc Emerg Med.

[31] Lindgren S., Karle H. (2011). Social accountability of medical education: aspects on global accreditation. Med Teacher.

[32] Bates J., Schrewe B., Ellaway R.H. (2019). Embracing standardisation and contextualisation in medical education. Med Educ..

[33] Nasr A.O., Lulic I., Mustafa M.T. (2023). Bringing critical emergency medicine, resuscitation and trauma education and training back to armed rivalry-affected community: why the conflict in Sudan matters?. Eur J Trauma Emerg Surg..

[34] Neutel E., Kuhn S., Driscoll P. (2023). Does participation in the European Trauma Course lead to new behaviours and organisational change? A Portuguese experience. BMC Med Educ.

