# Supplementary material for: The European Trauma Course: Transforming systems through training
Source: Resusc Plus. 2024 Mar 15;18:100599. doi: 10.1016/j.resplu.2024.100599 (PMC10955415; doi:10.1016/j.resplu.2024.100599)
Supplement: Supplementary data 1 [file mmc1.docx]

**ETC, Implementation and Effect at National Levels**

**Belgium**

By emphasizing training for both doctors and emergency care nurses, alongside other healthcare professionals, the ETC has fostered a cohesive, interdisciplinary approach to trauma care. This collaborative training model has enhanced communication and teamwork among different specialties, which is crucial for the effective management of trauma patients. The hands-on scenario approach allows participants to apply theoretical knowledge to practical situations, improving their skills in patient assessment, decision-making, and emergency procedures under pressure. This direct application of skills to realistic scenarios bridges the gap between theory and practice, most likely leading to improved patient outcomes. By integrating the ETC framework into daily clinical practice, major trauma centres have standardised patient care protocols. This standardisation ensures consistency in the management of trauma patients, from prehospital care to in-hospital treatment, across different levels of trauma centres. Uniform protocols, such as massive transfusion protocols and standardized trauma protocols, enhance the quality of care and patient safety.

Through the exchange of expertise and knowledge among ETC faculty and participants, the course contributes to the continuous improvement of trauma care practices. In Belgium. This culture of learning and adaptation leads to the evolution of more effective and efficient trauma care strategies.

The implementation of the ETC across Belgium has led to uniformity in the management of major trauma patients, not just within individual hospitals but across the entire healthcare system. This nationwide approach to trauma education and care ensures that all trauma victims receive a high standard of care, irrespective of where they are treated. The ETC's influence extends beyond hospital walls, affecting prehospital management and ultimately contributing to an improved chain of survival for trauma victims.

**Germany**

Since hosting its inaugural ETC in Mainz in 2008, Germany has expanded to 11 course centres, coordinating activities at approximately 25 venues, primarily in major trauma centres. The ETC in Germany follows a standardised programme to accommodate variances in trauma care practices across different centres and is open to various healthcare professionals, acknowledging the vital roles that healthcare professionals play in both prehospital and early in-hospital trauma management. While the participation of paramedics and nurses has been minimal, and the integration of TSP roles has been sporadic and diverse, ETC training is a recognised training programme for the accreditation of major trauma centres. Owing to the high demand, the prospect of implementing a shortened 2-day ETC program complemented with e-learning is being considered as a prehospital ETC variant to holistically address all facets of initial trauma care within the Emergency Medical Services (EMS). Notably, the adoption of ETC principles in regular trauma team training across many emergency departments in the country is a testament to the programme’s effectiveness and adaptability.

**Austria**

The ETC has been a prominent feature of Austria’s trauma care training since 2008, facilitated by ‘ETC-Austria’ since 2014. The model of a single course centre operating in 25 locations has been adopted successfully and has hosted over 100 courses.

ETC-Austria has incorporated local adaptations such as integrating human factor group exercises and modified scenarios addressing prehospital care. Additionally, it undertook pilot projects such as the Traumatic Cardiac Arrest (TCA) scenario, which was later adopted by the ETCO.

Initially exclusively open to physicians, the courses were opened to nurses and paramedics in 2009 after recognising their crucial roles in trauma teams. Austria aims to provide 10–15 courses per year across various hospitals.

**Italy**

In Italy, the ETC has been hosted at 15 different venues. These courses are primarily organised by major trauma centres and target medical doctors and nurses who are part of their trauma teams. The candidates were from diverse backgrounds, including anaesthesia, emergency medicine, general surgery, and emergency room nursing.

While Italy has retained the original ETC structure, there have been slight modifications to the handover process and Major Haemorrhage Protocol to cater for local requirements. The courses are conducted in Italian; there is a notable demand for an Italian language manual to enhance comprehension and serve as an easy accessible reference guide.

ETC training is recognised for trauma centre accreditation in Italy, and the Italian faculty aims to establish at least one-course centre in every region.

**Slovenia**

In Slovenia, the European Trauma Course (ETC) is predominantly based at a single centre and has seen specific adaptations to suit local clinical practices, including the early adoption of supraglottic airway devices and the utilization of intraosseous access. Despite its focus on doctors, there has been recognition of the need to include registered nurses in the training to reflect their crucial role in trauma care. Plans for future development include integrating the Trauma Support Practitioner Programme and engaging with the military sector.

Since the ETC's introduction, Slovenia has experienced significant improvements in patient management practices. A notable shift has been the implementation of the ABCDE approach across all patient care, not just trauma cases, promoting a consistent and structured assessment method across medical teams. Innovations such as the '5-second round' for immediate patient evaluation and 'STOP procedures' for strategic planning have streamlined patient care processes.

Moreover, an increased emphasis on non-technical skills, especially closed-loop communication, has been pivotal in enhancing team interactions, thereby improving both patient safety and care efficiency.

Overall, the ETC has profoundly impacted Slovenia's approach to patient management, fostering a more systematic and unified strategy across healthcare professions and significantly boosting communication and safety in patient care.

**Malta**

Since hosting its inaugural pilot ETC in November 2006, Malta has incorporated the course into its trauma training. Functioning under the Malta Resuscitation Council and based in a single major trauma hospital due to the nation's compact size, the ETC has been recognised as the gold standard in major trauma training and team practices. The courses are delivered according to the most up-to-date standards, efficiently reflect local practices, and remain exclusive to medical doctors, especially from Emergency Medicine, Anaesthesia, and Surgical specialities. Fifteen years after its inception, the ETC has maintained its pivotal role in ensuring quality medical training in Malta, consequently facilitating optimal, safe, and best-practice management for major trauma patients.

**Croatia**

Since its introduction in 2010 with the first session in Rijeka, the European Trauma Course (ETC) has significantly transformed trauma care in Croatia. Rijeka has become the cornerstone for ETC's expansion, showcasing Croatia's dedication to advancing trauma education both nationally and regionally.

A notable achievement is the organization of ETC courses in General Hospital Dubrovnik, part of the EU-funded TELE.DOC project, which extended trauma training to medical professionals in Bosnia and Herzegovina, Montenegro, and Croatia. This initiative highlighted the ETC's structured approach to trauma care, integrating algorithmic strategies with practical scenarios. Feedback from participants underscored the program's profound impact on their professional growth, marking a pivotal shift in trauma education.

Despite the challenges posed by the COVID-19 pandemic, the commitment to trauma education remained unwavering in Croatia. Rijeka consistently hosted two ETC courses annually, with seven courses conducted between 2020 and the present, underlining the resilience of the Croatian ETC community.

Croatia boasts a growing cadre of Full Instructors, Instructor Candidates, and Instructor Providers, predominantly based in Rijeka and adept at conducting semiannual ETC courses. Since 2010, the initiative has trained 335 medical professionals in cutting-edge trauma care, leading to observable enhancements in polytrauma treatment outcomes. The successful implementation of ETC algorithms, even with minimal provider presence, evidences the program's efficacy in clinical practice.

Looking to the future, Croatia aims to broaden ETC's influence within the country and the wider Southwestern European region. Plans include deploying mobile ETC faculties to educate new providers in various locales, thereby cultivating widespread interest and proficiency in trauma care. This strategic expansion promises to further elevate the standard of trauma treatment, making a lasting impact on the region's healthcare landscape.

**Portugal**

Since its introduction in Portugal in October 2009, the European Trauma Course (ETC) has been operational from three main centres: Porto, Coimbra, and Lisbon. Each centre, with its unique contributions and coordinators, has adapted the ETC to local needs, including a supportive programme for Instructor Candidates featuring mentoring and a digital platform with skills videos. Primarily attended by medical doctors and nurses, the programme may extend to Trauma Support Practitioners in the future.

Key changes in practice following the ETC's implementation include (34):

Individual Behavioural Changes: A notable 94% of participants reported that the ETC altered their approach to trauma care, with 71.4% experiencing significant behavioural changes, indicating a profound impact on clinical practices.

Non-Technical Skills (NTS): The ETC has positively influenced NTS, such as communication, prioritisation, and teamwork, with variations based on participants' leadership status and experience.

Organisational and National Impact: ETC instructors have played a pivotal role in adopting new institutional materials and therapeutic standards, suggesting their significant influence on organisational changes. The programme has encountered facilitators like confidence in the ETC methodology and challenges, including personality traits and a scarcity of ETC practitioners. Notably, the integration of the ETC into the Portuguese College of Anaesthesiology's resident program and the prioritisation of the National Trauma Registry underscore its positive impact at organisational and national levels.

These developments reflect the ETC's substantial role in enhancing trauma care quality in Portugal, demonstrating its effectiveness in fostering both individual and organisational advancements.

**Finland**

Since its launch in 2011 by the Finnish Trauma Association, the ETC has influenced trauma care practices in Finland. Expanding from an annual course to four or five yearly sessions across four centres, the program has maintained fidelity to the original ETC format, allowing for some local adaptations, such as the inclusion of nurses and paramedics as participants. The ETC's integration into Finland's specialist training programs for disciplines such as orthopedic surgery and acute care medicine illustrates its role in shaping medical education. This approach aligns with Finland's high standards in healthcare education and addresses the growing demand for trained professionals in trauma care. Challenges related to maintaining educational quality and expanding instructor numbers are acknowledged as the ETC's influence grows. Despite these challenges, the ETC's principles, including the primary survey and emphasis on communication and teamwork, have been incorporated into clinical practice and hospital protocols, reflecting a measured and pragmatic approach to improving trauma care in Finland.

**United Kingdom**

Launched in 2008, just a few months after its inception in Belgium, the ETC in the United Kingdom has become a highly regarded trauma training course for specialities and is accredited for specialist training. The ETC network has grown to 19 course centres scattered across the UK and Ireland, and in the last 5 years, nearly 2,500 candidates have completed the course.

The UK presents a unique candidate demographic when observed against the European backdrop, with 68% of candidates being emergency physicians, reflecting the nation’s well-structured speciality in emergency medicine. Anaesthesia and intensive care professionals followed, accounting for 24% of participants, while surgical specialists, such as those from trauma and orthopaedics, stood at only 5%, highlighting a minority representation in ETC participation. Non-medical professionals, such as trauma nurses and operating department practitioners, represented over 2% of the course candidates.

The concept of a true team approach, including emergency department nursing staff, culminated in the introduction of the role of the TSP in 2015. The TSP’s role included scribing, ensuring detailed, real-time clinical documentation, and acting as a hands-on clinical assistant. After comprehensive pilot studies and validation in Germany and the Netherlands, the ETCO Board approved the inclusion of TSPs as an optional course element by the end of 2022.

**Romania**

The ETC has become firmly established in Romania, with two centres in Targu Mures and Iasi. Romania's approach to Emergency Medicine features a comprehensive 5-year residency program with stringent parameters; as a result, all Romanian Emergency Medicine physicians can perform Rapid Sequence Intubation. This robust training has led to a scenario in which the airway clinician in a trauma situation is an Emergency Medicine specialist. Furthermore, the prehospital care framework in Romania has evolved to a point where intubation can be initiated even before the patient reaches the hospital, contingent upon the presence of a qualified Emergency Medicine physician within the EMS team.

ETC training also incorporates the disciplines of Anaesthesia and Intensive Care, Orthopaedics, General Surgery, Neurosurgery, Thoracic Surgery, and even General Practitioners equipped with competencies in prehospital Emergency Medicine.

Future effort should focus on increasing the number of instructors and the annual number of courses while investing in state-of-the-art training materials, with a special emphasis on high-fidelity manikins. There is also a strategic directive to bolster the number of course directors and extend expansion into neighbouring territories, such as Ukraine, the Republic of Moldova, and Albania.

**Netherlands**

In the Netherlands, the inaugural ETC was organised in 2017, originating from a collaborative effort between the Erasmus MC in Rotterdam and the Amsterdam UMC. The course is open to candidates and TSPs, with roles aligned with the actual functions performed during routine clinical care. Typically, candidate positions are filled by physicians with at least a few years of experience in trauma care, whereas nurse anaesthetists or ER nurses usually occupy TSP positions.

After thorough consideration, a decision was made to structure the Dutch ETC program within a national foundation. The board of this foundation boasts representatives from all course centres and all relevant national societies, including anaesthesia, emergency medicine, and trauma surgery.

Currently, three courses per year are held across two centres, with a plan to establish a third course centre and, through the expansion of faculty, to establish a self-supporting program. The goal is to teach 8–10 courses per year across 4–5 course centres, providing adequate capacity to train the majority of Level 1 and Level 2 clinicians and nurses while also accommodating some international candidates.

**Sweden**

The ETC operates primarily in Clinicum in Östergötland, which encompasses three central Swedish locations: Linköping, Norrköping, and Motala. Launched in 2014, two annual courses were rotated between these locations based on their availability. Given their international faculty and audiences, courses are conducted in English. The participants were predominantly emergency medicine, anaesthesia, and intensive care physicians. Surgical participants were scarce, which is attributed to the strong ATLS foothold in Sweden. While language barriers and unfamiliar leadership roles deter nurses, few have successfully completed the course. Recent courses have incorporated nurses into active scenarios, increasing the realism of the training. Their role in feedback and assessment is invaluable.

Local adjustments to the curriculum arose due to ambulances lacking intubation capabilities, often relying on iGel or seeking anaesthesiologist assistance. National spinal motion guidelines exclude hard cervical collars and limit spinal board use to short durations. Future objectives include broadening the course's geographical reach, particularly in Sweden's northern regions.

**Middle East & North Africa (MENA Region)**

The ETC found its way into the MENA region and was first introduced in Cairo, Egypt, in 2009. Subsequently, it reached countries such as Saudi Arabia, Sudan, Jordan, the UAE, and Tunisia until 2016. Various directors have pioneered these initiatives, embracing the course's unique approach to trauma education. However, substantial challenges arose primarily from staunch competition with the ATLS course, which is embedded and widely recognised in the region. Financial constraints, notably regarding sponsorships, manikins, and simulator costs, and operational challenges, such as managing a multiple-day course and dealing with high instructor-to-candidate ratios, further complicate the establishment of the ETC. Minor adaptations, such as a presentations on trauma biomechanics, retaining the initial Head-CT lecture and secondary survey sessions, helped to better tailor the course to regional needs. A set of broad suggestions and region-specific strategies revolving around enhancing affordability, expanding reach, forging collaborations, acquiring legal recognition, and instilling quality assurance mechanisms has been proposed to navigate through extant challenges and fortify the ETC's presence in the MENA region.

**Brazil**

Brazil is currently involved with the ETC at a single location in Campinas, with additional courses planned for Brazil. Brazil has an extensive history of trauma courses, notably the ATLS program, which has been running since 1989 and hosts over 100 courses annually across 20 centres. Establishing adequate local ETC faculty to allow self-sufficiency is challenging. Recent courses have included nurses experienced in trauma care and a senior medical students, a strategy that proved enriching and paved the way for potentially attracting more candidates in the future. Looking ahead, Brazil aims to expand its ETC offerings, with four courses scheduled for June and an additional two to four planned for another city. The aspiration is to offer 10 courses per year, typically organising 2 consecutive courses in various cities to facilitate the creation of additional centres. Although the Brazilian program is relatively nascent, it has already made a significant impact by integrating team training and non-technical skills (NTS) instruction into certain medical school curricula.

**Poland**

The ETC marked its debut in Poland in 2009, with courses being organised across three cities: Wrocław, Łódź, and Kraków. Between 2018 and 2023, the Medical University of Wrocław, supported by a grant from the European Union and Polish Ministry of Health, successfully conducted over 10 courses. Remarkably, these sessions were offered free of charge to the participants, a factor that significantly boosted the involvement of doctors in hospital emergency departments. The focus on non-technical skills, team management, and trauma team roles has led to a heightened interest in ETC, with requests for new courses emerging from both individuals and hospital administrators.

However, one limitation was the exclusive inclusion of doctors, as the project did not include other medical personnel. Despite this, the ETC is highly regarded in Poland and addresses crucial patient safety concerns. In the future, there are plans to diversify the participant base to include allied healthcare professionals who are actively engaged in trauma care.

In terms of course delivery, the ETC in Poland was conducted entirely in Polish, with the course materials translated accordingly. The sole exception is textbooks, which remain in English, posing challenges for those seeking comprehensive preparation but requiring greater proficiency in medical English.

**Hungary**

After experiencing an increasing demand for ETC courses in Hungary, this year marked the opening of a second centre. In addition to the well-established Pecs Centre, ETC courses are now available in Budapest. The two centres collectively offer four courses per year, conducted in both English and Hungarian. The participants primarily consisted of doctors from various specialities, predominantly emergency medicine and anaesthesia, with a few representatives from trauma surgery backgrounds and paramedics.

The Hungarian Society of Emergency Medicine has integrated ETC into its curriculum, requiring a certificate for the completion of speciality training. Hungary has closely adhered to the original ETC format, making minimal local adaptations such as a modified major haemorrhage protocol.

Future plans encompass the exploration of additional potential course centres, the development of more Hungarian-speaking faculty, and the inclusion of nurses and trauma support practitioners in training.
